# Association Between Serum Leucine and NT-proBNP Levels in Relation to Fragmented QRS: A Multiomic Analysis of the HOZUGAWA Cohort

**DOI:** 10.3390/nu18142271

**Published:** 2026-07-11

**Authors:** Kunimasa Yagi, Hiroshi Okada, Masahide Hamaguchi, Noriyuki Kitagawa, Yoshitaka Hashimoto, Hideki Origasa, Michiaki Fukui

**Affiliations:** 1 Department of Internal Medicine, Kanazawa Medical University Hospital, 1-1 Daigaku, Uchinada, Kahoku 920-0293, Japan; 2 Department of Endocrinology and Metabolism, Graduate School of Medical Science, Kyoto Prefectural University of Medicine, Kyoto 602-8566, Japan; conti@koto.kpu-m.ac.jp (H.O.); mhama@koto.kpu-m.ac.jp (M.H.); michiaki@koto.kpu-m.ac.jp (M.F.); 3 Department of Diabetology, Kameoka Municipal Hospital, Kyoto 621-8585, Japan; nori-kgw@koto.kpu-m.ac.jp; 4 Department of Diabetes and Endocrinology, Matsushita Memorial Hospital, Moriguchi 570-8540, Japan; y-hashi@koto.kpu-m.ac.jp; 5 Data Science and AI Innovation Research Promotion Center, Institute of Statistical Mathematics, Shiga University, Hikone 525-0034, Japan; origasahideki@gmail.com

**Keywords:** BCAA, leucine, electrocardiography, fragmented QRS, cardiac fibrosis, NT-proBNP, heart failure, HFpEF

## Abstract

Background/Objectives: Branched-chain amino acids (BCAAs) may play a protective role in the progression of heart failure; however, controversial results also exist. This study investigates the association between fasting serum BCAA levels and plasma NT-proBNP concentrations in individuals with fragmented QRS (fQRS) on ECGs within the HOZUGAWA health-checkup cohort in Japan, offering insights into cardiac health. Methods: This analysis included 252 participants who attended health check-ups. Fasting blood samples were analyzed using a standardized laboratory test. Unitless internal-standard-normalized relative peak-area ratios of BCAAs and selected amino-acid-related organic acids were measured and log-transformed for analysis. Results: NT-proBNP levels did not differ significantly between individuals with and without fQRS. Among those with fQRS, higher levels of BCAAs and 2-hydroxybutyric acid (2-HB) were associated with lower NT-proBNP levels: leucine (r = −0.38, *p* = 0.0001), while valine (r = −0.28, *p* = 0.0053) and isoleucine (r = −0.21, *p* = 0.041); 2-HB (r = −0.21, *p* = 0.039). After adjustment for age, sex, BMI, and estimated glomerular filtration rate (eGFR) (*n* = 252), leucine remained inversely associated with NT-proBNP in individuals with fQRS (r = −0.28, *p* = 0.0072) and positively associated in those without fQRS (r = 0.23, *p* = 0.0048). fQRS showed an interaction with leucine levels regarding NT-proBNP levels. Conclusions: Associations between relative leucine abundance and NT-proBNP levels varied by fQRS status, with significant inverse associations observed only among participants with fQRS, suggesting that lower relative BCAA abundance may be associated with markers of cardiac dysfunction and that leucine-related metabolic signatures may be linked to NT-proBNP levels in individuals with fQRS. Additional longitudinal and mechanistic studies are necessary to confirm these findings, particularly in individuals with cardiac fibrosis. Further research is warranted to explore therapeutic implications.

## 1. Introduction

In recent decades, the widespread adoption of Westernized diets and accelerated population aging have contributed to increased rates of diastolic dysfunction and heart failure with preserved ejection fraction (HFpEF) [1,2], a major subtype of congestive heart failure (CHF). Early identification of individuals at high risk is essential, as timely intervention may help reduce the growing burden of CHF.

Fragmented QRS (fQRS) on electrocardiography (ECG) serves as a noninvasive marker of myocardial fibrosis and structural damage and is associated with adverse cardiovascular outcomes [3]. Among patients with diabetes mellitus (DM), fQRS is also linked to diastolic dysfunction [4], indicating its potential as an early indicator of subclinical myocardial injury. Because fQRS assessment does not require imaging modalities, it offers a practical tool for identifying individuals at increased risk of CHF.

In addition to electrocardiographic markers, metabolic alterations have been implicated in the development and progression of heart failure. Among these, branched-chain amino acids (BCAAs), including leucine, isoleucine, and valine, are involved in metabolic signaling pathways that regulate protein turnover and energy metabolism.

Previous experimental and clinical studies have suggested both potentially beneficial [5,6] and detrimental [7,8,9,10] effects of BCAAs on cardiac remodeling and heart failure. However, the relationship between circulating BCAA levels and early cardiac dysfunction in community-dwelling individuals remains incompletely understood, particularly in the presence of myocardial fibrosis.

To address this knowledge gap, the HOZUGAWA study, a Japanese health-checkup cohort characterized by standardized fasting blood sampling and comprehensive phenotyping, offers a unique opportunity [11,12,13]. Utilizing a unified gas chromatography–mass spectrometry (GC/MS) platform, this cohort enables consistent profiling of amino acids and related metabolites. This resource supports the investigation of metabolic signatures associated with early cardiac dysfunction in a community-based population.

A hypothesis-driven analysis was performed to assess whether fasting branched-chain amino acids (BCAAs) and selected BCAA-adjacent or amino acid–related metabolites are associated with plasma N-terminal pro–B-type natriuretic peptide (NT-proBNP) levels, which serve as a marker of heart failure risk. This methodology aims to clarify the contribution of BCAAs and related metabolic pathways to subclinical myocardial injury in the early stages of CHF.

## 2. Materials and Methods

### 2.1. Study Design and Ethics

We conducted a cross-sectional analysis within the HOZUGAWA health checkup cohort at Kameoka Municipal Hospital in Kyoto, Japan. Throughout the study period, we enrolled all consecutive adult participants who underwent routine health checkups and satisfied the eligibility criteria. Standardized procedures comprised overnight fasting, venipuncture, and anthropometric measurements. The study protocol received approval from the Ethics Committee of Kyoto Prefectural University of Medicine (approval No. ERBC1503) and adhered to the Declaration of Helsinki as revised in 2013. Written informed consent was obtained from all participants.

### 2.2. Participants

The enrollment period extended from 7 July 2020, to 27 February 2024. Eligibility criteria were as follows: (i) availability of an overnight fasting serum sample processed for metabolomics and NT-proBNP; (ii) documentation of core covariates, including age, sex, BMI, and eGFR; and (iii) ECG recordings without evidence of arterial fibrillation. To maintain analytic consistency and ensure reliable classification, participants were excluded from the analysis using complete-case analysis if fasting serum samples were missing.

### 2.3. Plasma NT-proBNP Measurement and Thresholds

Plasma NT-proBNP levels were measured using a chemiluminescent immunoassay (Lumipulse Presto NT-proBNP, FUJIREBIO, Tokyo, Japan). A NT-proBNP level > 55 pg/mL is regarded an upper standard limit, and a BNP level ≥ 125 pg/mL is regarded as elevated, supporting a diagnosis of heart failure [14,15].

### 2.4. ECG Recording and fQRS Criteria

A resting 12-lead ECG was recorded on admission in the supine position using an FCP-7431 ECG machine (Fukuda Denshi Co., Ltd., Tokyo, Japan) with standard settings: filter range 0.16–100 Hz, AC filter 60 Hz, paper speed 25 mm/s, and calibration 10 mm/mV. Each ECG was analyzed by one cardiologist and confirmed by another, both blinded to clinical and laboratory data. The concordance rate for detecting fQRS was 97%, consistent with previous studies [16,17]. fQRS was defined as described by Das et al. [18]. In patients without bundle branch block, fQRS was identified as RSR′ patterns, including an additional R wave (R′), notching of the R or S wave, or more than one R′ in at least two contiguous leads corresponding to a major coronary artery territory or left ventricular segment. In patients with bundle branch block (QRS duration ≥ 120 ms by standard ECG criteria), fQRS was defined as RSR′ patterns with or without a Q wave, including more than two R waves (R′), more than two notches in the R wave, or more than two notches in the upstroke or downstroke of the S wave in at least two contiguous leads corresponding to a major coronary artery territory [3]. fQRS was considered present when these findings were observed in two or more contiguous anterior, lateral, or inferior leads.

### 2.5. Serum Metabolomics (GC/MS with Solid-Phase Dehydration Derivatization)

Fasting serum organic acids and amino acids were profiled by GC/MS after solid-phase dehydration derivatization using an inline platform (SPL M100; AiSTI SCIENCE, Wakayama, Japan). This workflow is based on solid-phase analytical derivatization approaches developed for GC/MS metabolomics and supports harmonized measurement of chemically diverse metabolites on a unified analytical system [11]. All serum samples were collected under standardized overnight-fasting conditions and stored until analysis; despite the multi-year enrollment window, metabolomics measurements for this study were performed in a consolidated analytical campaign using identical SOPs and instrument configuration. The analytical workflow and internal-standard normalization strategy have been described previously and applied in HOZUGAWA studies [11,12,13].

Briefly, extracted aliquots (50 µL) were loaded onto a stacked ion-exchange FlashSPE ACXs cartridge, washed (acetonitrile:water = 1:1), dehydrated with acetonitrile, and nitrogen-dried on the cartridge. For organic acids and amino acids, on-cartridge methoximation (methoxyamine·HCl/pyridine) was followed by trimethylsilylation (MSTFA/hexane). Derivatized metabolites were eluted in hexane and injected via a programmable-temperature large-volume injector (LVIS250; AiSTI SCIENCE) onto a VF-5 ms capillary column (30 m × 0.25 mm i.d., 0.25 µm film; Agilent Technologies, Santa Clara, CA, USA). Data were acquired in scan mode (*m*/*z* 70–600).

Metabolite identification was performed using MS-DIAL (v4.9) [19] against an in-house library that incorporates retention indices and electron ionization (EI) mass spectra. Quantification was based on internal-standard-normalized relative peak-area ratios (analyte/IS) rather than absolute concentrations. For each metabolite, the peak area of a predefined analyte-specific extracted quantifier ion from the deconvoluted GC/MS chromatographic peak was divided by the peak area of the quantifier ion of the corresponding internal standard selected by metabolite class. L-norleucine was used as the internal standard for amino acids, including leucine, isoleucine, valine, alanine, and glycine, whereas adipic acid was used for organic acids, including 2-hydroxybutyrate. Total ion chromatogram areas were not used for quantification. The resulting metabolite values were dimensionless and represented semi-quantitative relative abundances. Because authentic calibration standards and metabolite-specific calibration curves were not generated for each analyte, the values were not converted to standard concentration units such as umol/L. For this hypothesis-driven secondary analysis, leucine (Leucine_2TMS), Isoleucine (Isoleucine_2TMS), Valine (Valine_2TMS), 2-hydroxybutyrate (2-Hydroxybutyric acid_2TMS), Alanine (Alanine _2TMS), and glycine (Glycine_3TMS) were selected a priori as primary exposures (Appendix A).

The metabolites included in this hypothesis-driven analysis were selected a priori based on their established biological relevance to BCAA metabolism and cardiovascular physiology. Leucine, isoleucine, and valine were identified as primary metabolites of interest due to their documented roles in myocardial energy metabolism, protein turnover, and the pathophysiology of heart failure. Alanine was selected because it participates in the glucose–alanine cycle and reflects amino acid–mediated energy metabolism, whereas glycine was included because of its role in one-carbon metabolism, antioxidant defense, and cardiovascular health. Together with 2-HB, these metabolites were chosen to capture metabolic pathways adjacent to BCAA metabolism that may be relevant to myocardial stress and remodeling.

Total BCAAs were calculated as the sum of the internal-standard–normalized relative peak-area ratios for leucine, isoleucine, and valine before natural-log transformation. As metabolite measurements were normalized to internal standards and reported as unitless relative peak-area ratios rather than absolute concentrations, the results should be interpreted as relative metabolomic abundance rather than circulating metabolite concentrations. The internal standard was L-norleucine for the BCAA and other amino-acid variables and adipic acid for 2-HB.

### 2.6. Statistical Analysis

All statistical tests were two-sided, and *p*-values < 0.05 were considered statistically significant. Analyses were performed using R version 4.3.0 with RStudio version 2024.09.1+394 (R Foundation for Statistical Computing, Vienna, Austria) and JMP Student Edition version 18.2.0 (SAS Institute Inc., Cary, NC, USA). Analyses were performed on a Macintosh computer. ChatGPT 5.5 was used to check statistical code; all code and outputs were independently reviewed and verified by the authors.

Categorical variables were compared between the subjects with fQRS and those without fQRS using Pearson’s χ^2^ test; Fisher’s exact test was used for 2 × 2 tables when expected cell counts were <5. Continuous variables were summarized as mean ± standard error. Group comparisons used Welch’s *t*-test for approximately normally distributed variables and the Wilcoxon rank-sum test otherwise. Missing data were handled using listwise deletion, and no multiplicity adjustment was applied because the analysis focused on BCAAs and a priori selected metabolites related to BCAA or amino acid metabolism, including 2-HB, alanine, and glycine.

To evaluate associations between fasting serum metabolites and plasma NT-proBNP levels, we fit multivariable-adjusted correlation analyses with NT-proBNP levels as the dependent variable. BCAAs and selected BCAA-adjacent or amino-acid–related metabolites values were natural-log transformed to reduce right-skewness and improve model stability. When zero values were present, a small offset (one-half of the minimum positive value for the variable) was added prior to log transformation. Primary models were adjusted for age (years), sex (male), eGFR (mL/min/1.73 m^2^), and body mass index (BMI; kg/m^2^). Covariate selection for the primary models was informed a priori. Age, sex, eGFR, and BMI constituted the core (minimal) adjustment set for the primary association models. Data regarding dietary protein intake, physical activity, medication use, and other lifestyle-related factors were not systematically collected for all participants and therefore could not be incorporated into the primary adjustment models.

## 3. Results

**Figure 1:**
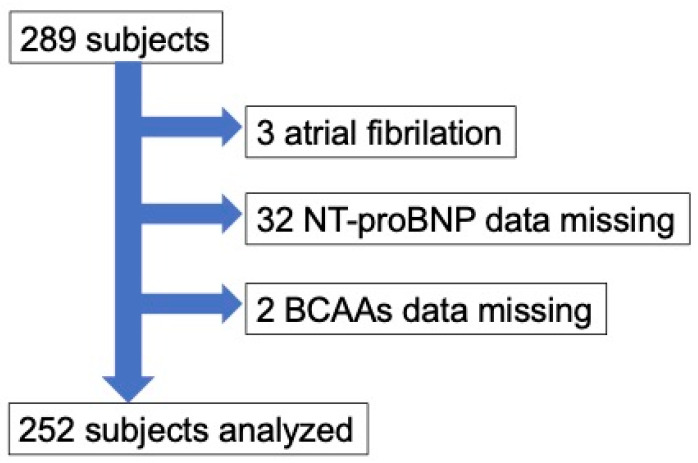
Study workflow.

Figure 1 presents the participant flow and sample sizes included in the analysis. A total of 252 participants with complete data on leucine, isoleucine, valine, 2-HB, alanine, and glycine were included in the primary regression analyses.

Study population flow diagram. Among 289 enrolled subjects, 252 were included in the final analysis after exclusion of participants with atrial fibrillation (n = 3), missing NT-proBNP data (n = 32), and missing BCAA measurements (n = 2).

Table 1 summarizes the basic characteristics stratified by fQRS. Subsequent sections detail group comparisons and correlation findings. Comparison between fQRS groups indicated that participants with fQRS were more likely to be male than those without fQRS. No significant differences in plasma NT-proBNP levels and serum BCAAs and selected BCAA-adjacent or amino-acid–related metabolites were observed between the fQRS groups.

**Figure 2:**
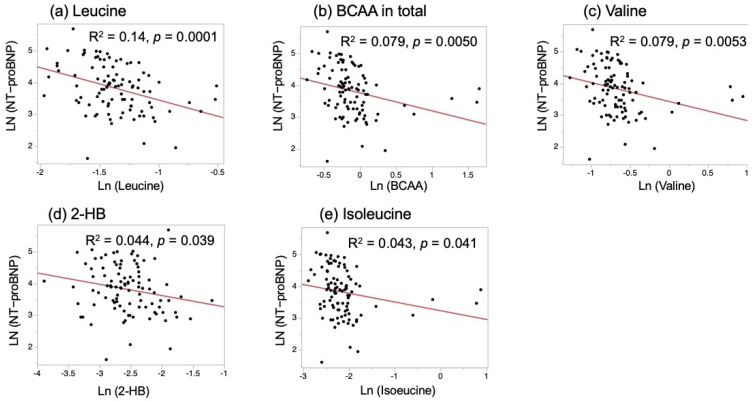
Relationship between relative serum levels of branched-chain amino acids (BCAAs) and selected amino-acid–related metabolites and plasma NT-proBNP levels in participants with fQRS. Scatter plots showing the associations between relative serum levels of (**a**) leucine, (**b**) total BCAAs, (**c**) valine, (**d**) 2-hydroxybutyrate (2-HB), and (**e**) isoleucine and plasma NT-proBNP levels among participants with fragmented QRS (fQRS). Linear regression analyses demonstrated significant correlations for all variables (R^2^ = 0.043–0.14, *p* < 0.05). Black dots represent individual study participants, and the red line indicates the fitted linear regression line.

When stratified by fQRS status, distinct patterns emerged. Among participants with fQRS, BCAA relative serum levels differed significantly between those with NT-proBNP levels greater than 55 pg/mL and those with levels at or below 55 pg/mL (Supplementary Figure S1). Among participants with fQRS, ln-transformed BCAA and 2-HB relative serum levels significantly inversely correlated with ln-transformed NT-proBNP levels (Figure 2, Table 2); leucine (r = −0.38, *p* = 0.0001), valine (r = −0.28, *p* = 0.0053), isoleucine (r = −0.21, *p* = 0.041), and 2-HB (r = −0.21, *p* = 0.039). In contrast, these associations were not observed in participants without fQRS.

Metabolite values are internal-standard-normalized relative peak-area ratios and are therefore unitless; amino-acid variables were normalized to L-norleucine and 2-HB to adipic acid. The plotted metabolite variables were natural-log transformed and should be interpreted as semi-quantitative relative abundances rather than absolute serum con-centrations.

**Figure 3:**
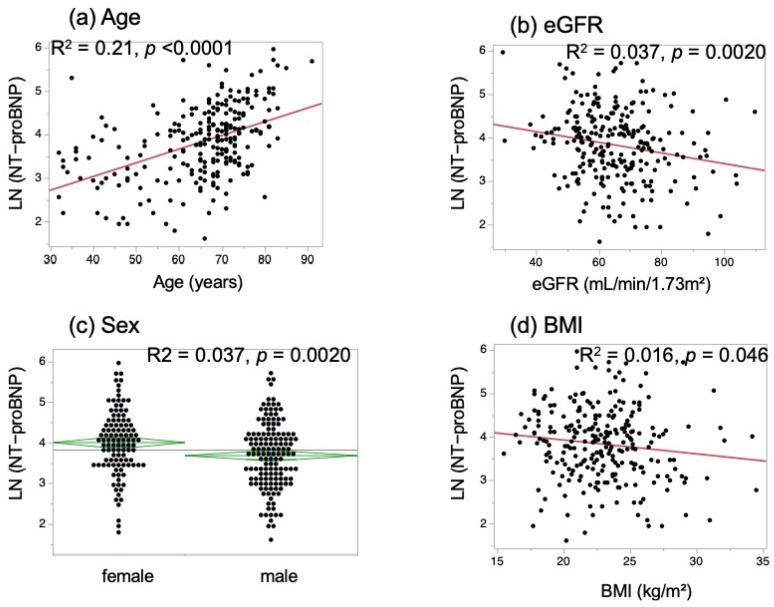
Factors significantly associated with NT-proBNP. Scatter plots illustrating the relationships between ln-transformed NT-proBNP levels and (**a**) age, (**b**) estimated glomerular filtration rate (eGFR), (**c**) sex, and (**d**) body mass index (BMI). Linear regression analyses showed that age, eGFR, sex, sex and BMI were significantly associated with ln-transformed NT-proBNP (R^2^ = 0.016–0.21, all *p* < 0.05). Black dots represent individual study participants, and the red line indicates the fitted linear regression line.

In correlational analyses, age, sex, eGFR, and BMI were significantly associated with NT-proBNP levels (Figure 3). After adjustment for age, sex, eGFR, and BMI (n = 252), partial correlation analyses revealed a significant negative association between ln-transformed leucine and NT-proBNP levels among participants with fQRS (*p* = 0.0072), indicating that higher leucine levels were associated with lower NT-proBNP concentrations (Table 3). Conversely, among participants without fQRS, a positive association was observed (*p* = 0.0048).

Furthermore, a multiple linear regression model was constructed with Ln (NT-proBNP) as the dependent variable and fQRS and Ln (Leucine) as independent variables (Table 4). Table 4-1 presents a model adjusted for age, sex, eGFR, and BMI, demonstrating a significant interaction term [fQRS(+)] × [ln(leucine) + 1.35147] (*p* = 0.0007). Table 4-2 further adjusts for diabetes mellitus, protein intake, skeletal muscle mass, and regular exercise; to mitigate collinearity, skeletal muscle mass replaces BMI. In this model, the interaction term [fQRS(+)] × [ln(leucine) + 1.35415] remains significant (*p* = 0.0043), supporting a significant interaction between fQRS and ln (leucine) with respect to ln (NT-proBNP).

## 4. Discussion

This secondary analysis of the Japanese HOZUGAWA health-checkup cohort identified an inverse association between relative leucine abundance and NT-proBNP levels in individuals with fQRS. The association persisted after adjustments for age, sex, BMI, and eGFR. Importantly, these associations were not observed in the overall study population and were largely restricted to participants with fQRS. Therefore, the findings should be interpreted as subgroup-specific observations rather than evidence of a generalized relationship.

First, because some previous studies have suggested potentially adverse cardiovascular effects of excessive BCAA accumulation, our findings warrant careful interpretation. The present findings do not support a detrimental association of higher relative leucine abundance with NT-proBNP levels within the exposure range observed in this cohort. Previous reports of BCAA toxicity most likely pertain to cases involving substantially higher exposures [9,10]. The observed discrepancies are attributable to variations in exposure range and clinical context rather than genuine contradictions.

Second, the subgroup-specific nature of the findings may provide an important clue to their biological interpretation. The findings raise the possibility that altered amino acid metabolism is linked to cardiac dysfunction in individuals with myocardial fibrosis. One possible interpretation is that myocardial fibrosis or related structural abnormalities influence the relationship between amino acid metabolism and cardiac stress biomarkers. In this context, relative circulating BCAA signals, particularly leucine-derived metabolomic measures, may reflect metabolic alterations associated with fibrotic myocardial remodeling. However, these observations should be interpreted with caution, as they may also be attributable to random variation inherent in subgroup analyses. Replication in independent cohorts is essential to validate these findings and clarify their biological significance.

To support this hypothesis, several experimental observations may help explain the observed association. Clinical and experimental studies have demonstrated that BCAA administration can enhance myocardial protein synthesis and shift myocardial phenylalanine balance from negative to neutral [5]. Oral BCAA supplementation in a Dahl salt-sensitive rat CHF model improved cardiac function and attenuated cachexia-related changes [6]. Whereas excessive BCAA accumulation has also been associated adversely with impaired cardiac function [7], altered insulin signaling [8], and myocardial fibrosis [9]. These findings suggest that the effects of BCAAs may depend on the underlying cardiac substrate, exposure level, and metabolic context. Our findings extend this literature by demonstrating that the association between circulating leucine and NT-proBNP varies by fQRS status, a marker of myocardial fibrosis. Because of the cross-sectional design, causality cannot be inferred, and reverse causation remains possible.

Third, leucine appeared to show a stronger association with NT-proBNP than other BCAAs under specific conditions. Leucine promotes myocardial protein synthesis, distinguishing it from isoleucine and valine. In rat heart models, leucine at 1 mM increased protein synthesis by approximately 25% and reduced degradation by 14–29%, thereby improving nitrogen balance [20]. In neonatal pig models, individual BCAAs activated translation initiation factors eIF4E-BP1 and eIF4G exclusively in the left ventricular wall, resulting in increased protein synthesis rates. Isoleucine and valine did not demonstrate similar effects [21]. These findings highlight leucine’s specificity among BCAAs and are consistent with the present results. Leucine also suppresses myocardial protein degradation. In isolated rat left atrial preparations, leucine at five times its physiological plasma concentration reduced protein degradation by 15–21%. Although this effect is less pronounced than that of insulin, it supports a leucine-specific role in protein turnover [22]. Furthermore, several preclinical studies [23,24,25,26] have demonstrated beneficial effects of leucine supplementation on cardiac structure and function. The present findings suggest that lower relative leucine abundance may reflect metabolic states associated with diminished leucine availability, although actual circulating leucine concentrations were not quantified in this study, potentially influencing cardiac stress responses. Although this interpretation remains speculative, the evidence supports a significant role for leucine in cardiac protein metabolism. However, the biological effects of leucine may not be uniform across different myocardial conditions.

In contrast, experimental evidence suggests that leucine may exert different effects in myocardium without overt fibrotic remodeling. BCAAs are recognized for their ability to activate mTOR pathway. Davoodi and Hutson demonstrated that increased leucine concentrations in BCATm-knockout mice induce cardiac hypertrophy through mTOR activation [27]. Similarly, Latimer et al. found that BCAA-enriched meals provided at the end of the active phase increase both cardiac mass and cardiomyocyte size via mTOR signaling [28]. Shende et al. observed that pressure overload-induced hypertrophy elevates atrial and brain natriuretic peptide levels, implicating mTOR signaling in this response [29]. These observations raise the possibility that the biological effects of leucine differ according to the underlying myocardial substrate.

Collectively, the present findings and prior experimental evidence indicate that the relationship between leucine metabolism and cardiac stress may be influenced by the underlying myocardial condition. Among individuals with fQRS, a marker of myocardial fibrosis, higher relative leucine abundance was associated with lower NT-proBNP levels. In contrast, experimental studies have shown that leucine can promote hypertrophic signaling via mTOR activation in other contexts. These results suggest that leucine may have distinct biological effects depending on the extent of myocardial remodeling.

Nevertheless, the current study employed a cross-sectional design, and the most robust associations were identified only in subgroup analyses. Furthermore, dietary leucine intake, supplementation, and longitudinal clinical outcomes were not evaluated. Consequently, these findings should be regarded as hypothesis-generating, and additional mechanistic and prospective studies are required to elucidate the role of leucine metabolism in cardiac remodeling and dysfunction.

### 4.1. Implications and Future Directions

The results indicate that leucine-related metabolomic signatures may be particularly relevant in subgroups defined by fQRS, rather than in the general population. The observed associations may partly reflect differences in dietary habits, physical activity, medication exposure, or metabolic health status that were not fully captured in the available dataset. Longitudinal studies using quantitative metabolite measurements are necessary to determine whether circulating leucine concentrations independently predict progression to CHF beyond established risk factors. Furthermore, emerging interventional studies suggest that dietary BCAA intake may influence heart failure progression. The relevance of BCAA restriction and supplementation, including leucine-focused strategies, should be established in rigorously controlled clinical settings.

### 4.2. Strengths and Limitations

This study demonstrates several strengths. The HOZUGAWA cohort is community-based and uses standardized overnight fasting sampling, thereby enhancing internal validity and supporting its applicability to real-world screening. Metabolite profiling was conducted using a unified GC/MS platform with solid-phase dehydration derivatization, thereby reducing analytical heterogeneity and enabling consistent comparisons across metabolite classes [11,30]. Metabolite identification employed MS-DIAL workflows for standardized deconvolution and annotation [19]. A hypothesis-driven analysis was performed, focusing on BCAAs, fQRS on ECG, and NT-proBNP levels, which reduced the multiplicity burden associated with untargeted screening.

Several limitations warrant consideration. The cross-sectional design precludes causal inference and does not establish temporality; reverse causation remains possible, including potential effects from treatment or dietary modification following diagnosis. Metabolite values for BCAAs and selected BCAA-adjacent or amino-acid-related metabolites were reported as unitless internal-standard-normalized relative peak-area ratios rather than absolute concentrations. Amino-acid variables were normalized to L-norleucine, whereas organic acids such as 2-HB were normalized to adipic acid. This semi-quantitative approach may limit comparability and clinical interpretability. These values should not be directly compared with standard concentration values from fully quantitative assays.

Residual confounding represents a significant limitation of this study. Dietary protein intake and physical activity are primary determinants of circulating amino acid profiles and may also influence NT-proBNP concentrations through their effects on body composition, skeletal muscle metabolism, and cardiovascular fitness. However, these variables were not systematically collected and thus could not be included in the primary adjustment models.

Furthermore, medication use, including treatments for hypertension, diabetes, dyslipidemia, and cardiovascular disease, may have influenced both amino acid metabolism and natriuretic peptide concentrations. Underlying metabolic conditions such as insulin resistance, metabolic syndrome, diabetes severity, frailty, and sarcopenia could also have contributed to the observed associations. Given that circulating branched-chain amino acid (BCAA) levels are closely related to nutritional status and skeletal muscle mass, variations in these factors may partially account for the associations observed between leucine-related metabolomic measures and NT-proBNP levels.

Although the primary models were adjusted for age, sex, body mass index (BMI), and kidney function, residual confounding from unmeasured lifestyle, nutritional, metabolic, and treatment-related factors cannot be excluded. Consequently, these findings should be interpreted as exploratory observational associations rather than as evidence of independent biological effects.

## 5. Conclusions

Within this Japanese health-checkup cohort, lower relative leucine abundance was associated with higher NT-proBNP levels among individuals with fQRS, whereas no significant association was observed in the overall population. These results indicate that leucine-related metabolic signatures may interact with phenotypes associated with myocardial fibrosis and support the hypothesis that the biological effects of leucine vary with myocardial substrate. Nevertheless, due to the cross-sectional study design and the subgroup-specific nature of these findings, validation in independent prospective studies is necessary.

## Figures and Tables

**Table 1:** Clinical Characteristics of the Study Population.

	fQRS (+)	fQRS (−)	*p* Value
N	97	155	
Age, years	66.9 ± 1.2	64.2 ± 1.0	0.072
Male sex, n (%)	65 (67%)	78 (50%)	0.0093
Systolic BP, mmHg	134.9 ± 1.7	134.9 ± 1.4	0.99
Diastolic BP, mmHg	80.9 ± 1.3	81.3 ± 0.9	0.83
Heart rate,/min	61.6 ± 1.0	62.7 ± 0.8	0.39
BMI, kg/m^2^	22.8 ± 0.3	23.0 ± 0.3	0.53
eGFR, mL/min	64.4 ± 1.3	65.8 ± 1.0	0.43
AST, U/L	24.3 ± 1.7	23.2 ± 0.6	0.47
ALT, U/L	23.9 ± 2.0	21.3 ± 0.8	0.17
LDL-C, mg/dL	125.9 ± 2.9	123.7 ± 2.2	0.55
TG, mg/dL	110.8 ± 6.9	105.1 ± 4.4	0.46
HbA1c, %	5.93 ± 0.07	5.98 ± 0.06	0.57
NT-proBNP	59.6 ± 4.7	66.9 ± 5.2	0.34
NT-proBNP > 55 pg/mL	38 (39%)	72 (46%)	0.26
NT-proBNP ≥ 125 pg/mL	9 (9%)	17 (11%)	0.67
Leuvine	0.27 ± 0.01	0.31 ± 0.04	0.32
Isoleucine	0.17 ± 0.03	0.19 ± 0.06	0.80
Valine	0.56 ± 0.04	0.74 ± 0.17	0.41
Alanine	0.66 ± 0.06	0.89 ± 0.21	0.39
Glycine	0.48 ± 0.02	0.54 ± 0.03	0.14
2-HB	0.086 ± 0.076	0.088 ± 0.080	0.71
ECG findings			
Blocks			
1′ AVB	3 (3.1%)	6 (3.9%)	0.75
RBBB & ICRBBB	15 (15.5%)	3 (1.9%)	<0.0001
LBBB & LAD	4 (4.1%)	6 (3.9%)	0.92
fQRS			
fQRS region			
Inferior leads	67 (69.1%)	N/A	N/A
Anterior leads	42 (43.3%)	N/A	N/A
Lateral leads	5 (5.2%)	N/A	N/A
Multiple regions	15 (15.5%)	N/A	N/A
fQRS morphologies			
Fragmented QRS	2 (2.1%)	N/A	N/A
rSr′	6 (6.2%)	N/A	N/A
Notched S	68 (70.1%)	N/A	N/A
RSR′	3 (3.1%)	N/A	N/A
Notched R	84 (86.6%)	N/A	N/A

Continuous variables are presented as the mean ± standard error, and categorical variables as number (percentage). Comparisons were made between participants with and without fragmented QRS (fQRS). Metabolite values are shown as internal-standard-normalized relative peak-area ratios and are therefore unitless; amino-acid variables were normalized to L-norleucine and 2-HB to adipic acid. They represent semi-quantitative relative abundances, not absolute serum concentrations. ALT, alanine aminotransferase; AST, aspartate aminotransferase; AVB, atrioventricular block; BMI, body mass index; BP, blood pressure; ECG, electrocardiography; eGFR, estimated glomerular filtration rate; fQRS, fragmented QRS; HbA1c, hemoglobin A1c; ICRBBB, incomplete right bundle branch block; LAD, left anterior fascicular block; LBBB, left bundle branch block; LDL-C, low-density lipoprotein cholesterol; N/A, not Applicable; NT-proBNP, N-terminal pro–B-type natriuretic peptide; RBBB, right bundle branch block; TG, triglycerides; 2-HB, 2-hydroxybutyrate.

**Table 2:** Correlation between ln-transformed NT-proBNP and ln-transformed BCAAs and related metabolic factors.

Group	BCAA	n	r	*p* Value
All	ln(Leucine)	252	−0.021	0.74
All	ln(Isoleucine)	252	−0.0027	0.97
All	ln(Valine)	252	−0.0040	0.95
All	ln(BCAA in total)	252	−0.0082	0.90
All	ln(Alanine)	252	0.058	0.36
All	ln(Glycine)	252	0.052	0.41
All	ln(2-HB)	252	−0.11	0.072
fQRS (+)	ln(Leucine)	97	−0.38	0.0001
fQRS (+)	ln(Isoleucine)	97	−0.21	0.041
fQRS (+)	ln(Valine)	97	−0.28	0.0053
fQRS (+)	ln(BCAA in total)	97	−0.28	0.005
fQRS (+)	ln(Alanine)	97	−0.051	0.62
fQRS (+)	ln(Glycine)	97	−0.098	0.34
fQRS (+)	ln(2-HB)	97	−0.21	0.039
fQRS (−)	ln(Leucine)	155	0.11	0.18
fQRS (−)	ln(Isoleucine)	155	0.11	0.16
fQRS (−)	ln(Valine)	155	0.11	0.19
fQRS (−)	ln(BCAA in total)	155	0.11	0.17
fQRS (−)	ln(Alanine)	155	0.10	0.20
fQRS (−)	ln(Glycine)	155	0.11	0.17
fQRS (−)	ln(2-HB)	155	−0.066	0.41

Correlation analyses between ln-transformed NT-proBNP (dependent variable) levels and ln-transformed branched-chain amino acids (BCAAs) and related metabolic factors (independent variables) in the overall population and stratified by fragmented QRS (fQRS) status. Correlation coefficients (r) and corresponding *p* values are shown. The metabolite variables were natural-log-transformed internal-standard-normalized relative peak-area ratios (unit-less); amino-acid variables were normalized to L-norleucine and 2-HB to adipic acid. BCAAs include leucine, isoleucine, valine, and total BCAAs. Related metabolic factors include alanine, glycine, 2-hydroxybutyrate (2-HB), and 3-hydroxybutyrate (3-HB). NT-proBNP, N-terminal pro–B-type natriuretic peptide; BCAAs, branched-chain amino acids; fQRS, fragmented QRS; ln, natural logarithm; 2-HB, 2-hydroxybutyrate.

**Table 3:** Adjusted correlations between NT-proBNP and ln-transformed BCAAs.

Group	BCAA	n	r	95% CI			*p* Value
All	ln(Leucine)	252	19.5	0.085	−	38.9	0.049
All	ln(Isoleucine)	252	6.46	−5.78	−	18.7	0.30
All	ln(Valine)	252	10.7	−4.84	−	26.2	0.18
All	ln(BCAA in total)	252	0.091	0.028	−	0.15	0.15
fQRS (+)	ln(Leucine)	97	−45.0	−77.4	−	−12.5	0.0072
fQRS (+)	ln(Isoleucine)	97	−10.30	−24.50	−	3.870	0.15
fQRS (+)	ln(Valine)	97	−21.4	−44.7	−	1.95	0.072
fQRS (+)	ln(BCAA in total)	97	−20.7	−42.7	−	1.30	0.065
fQRS (−)	ln(Leucine)	155	34.9	10.8	−	59.0	0.0048
fQRS (−)	ln(Isoleucine)	155	18.3	0.40	−	36.2	0.045
fQRS (−)	ln(Valine)	155	20.7	0.69	−	40.7	0.043
fQRS (−)	ln(BCAA in total)	155	23.4	2.92	−	43.8	0.025

Multivariable-adjusted correlation analyses between NT-proBNP levels (dependent variable) and ln-transformed branched-chain amino acids (BCAAs) (independent variables) in the overall population and stratified by fragmented QRS (fQRS) status. Analyses were adjusted for age, sex, estimated glomerular filtration rate (eGFR), and body mass index (BMI). Correlation coefficients (r), 95% confidence intervals (CI), and corresponding *p* values are shown. The BCAA variables were natural-log-transformed internal-standard-normalized relative peak-area ratios (unitless; normalized to L-norleucine). BCAAs include leucine, isoleucine, valine, and total BCAAs. BCAAs, branched-chain amino acids; BMI, body mass index; CI, confidence interval; eGFR, estimated glomerular filtration rate; fQRS, fragmented QRS; ln, natural logarithm; NT-proBNP, N-terminal pro–B-type natriuretic peptide.

**Table 4:** Interaction between fQRS and Ln (Leucine) for Ln (NT-proBNP).

1						
Factors	estimate	SE	95% CI			*p*-value
Age	0.031	0.0042	0.023	–	0.039	<0.0001
Sex	0.18	0.048	0.084	–	0.28	0.0003
[fQRS (+)] * [ Ln(Leucine) + 1.35147]	−0.51	0.15	−0.81	–	−0.22	0.0007
eGFR	−0.0037	0.0038	−0.011	–	0.0039	0.34
BMI	−0.012	0.015	−0.041	–	0.017	0.42
fQRS (+)	−0.027	0.046	−0.12	–	0.065	0.56
Ln(Leucine)	−0.070	0.16	−0.39	–	0.25	0.66
2						
Factors	estimate	SE	95% CI			*p*–value
Age	0.033	0.0051	0.023	–	0.043	<0.0001
[fQRS (+)] * [ Ln(Leucine) + 1.35415]	−0.48	0.16	−0.80	–	−0.15	0.0043
DM (+)	−0.31	0.13	−0.57	–	−0.047	0.021
Sex	0.12	0.085	−0.043	–	0.29	0.14
Protein intake	−0.0020	0.0021	−0.0060	–	0.0021	0.34
eGFR	−0.0030	0.0040	−0.011	–	0.0050	0.46
Skeltal Muscle Mass	−0.0073	0.016	−0.038	–	0.024	0.64
fQRS (+)	−0.012	0.049	−0.11	–	0.085	0.81
Ln (Leucine)	−0.021	0.18	−0.38	–	0.34	0.91
Regular exercise (+)	0.0027	0.13	−0.26	–	0.26	0.98

A multiple linear regression model was developed with ln(NT-proBNP) as the dependent variable, incorporating fQRS status, Ln (leucine), and their interaction term. Table 4-1 presents the interaction model adjusted for age, sex, estimated glomerular filtration rate (eGFR), and body mass index (BMI). Table 4-2 includes additional adjustments for potential determinants of circulating leucine levels, such as diabetes mellitus, protein intake, skeletal muscle mass, and regular exercise. To avoid collinearity, skeletal muscle mass was used instead of BMI. The interaction term was calculated using mean-centered Ln (leucine). Protein intake was quantified in grams per day. Skeletal muscle mass was assessed by bioelectrical impedance analysis (InBody) and reported in kilograms. Regular exercise was defined as participation in at least one hour of exercise per session on a minimum of two days per week. Abbreviations: BMI, body mass index; CI, confidence interval; DM, diabetes mellitus; eGFR, estimated glomerular filtration rate; fQRS, fragmented QRS; ln, natural logarithm; NT-proBNP, N-terminal pro–B-type natriuretic peptide; SE, standard error. * Interaction term between fQRS (+) and mean-centered ln(leucine).

## Data Availability

Deidentified individual participant data reported in this article will be shared upon reasonable request. Data will be available beginning three months after publication and for up to five years. Researchers with a methodologically sound proposal supporting an approved study may request access by contacting the corresponding author. Data will be provided upon approval of the proposal and completion of a data use agreement.

## Supplementary Materials

The following supporting information can be downloaded at: https://www.mdpi.com/article/10.3390/nu18142271/s1, Supplementary Figure S1. Differences in serum BCAA and selected amino-acid–related metabolites according to NT-proBNP levels in participants with fQRS.

## Abbreviations

The following abbreviations are used in this manuscript:

BCAAs	Branched-chain amino acids
fQRS	fragmented QRS
NT-proBNP	N-terminal pro B-type natriuretic peptide
BMI	body mass index
eGFR	estimated glomerular filtration rate
2-HB	2-hydroxybutyric acid
CHF	congestive heart failure
HFpEF	heart failure with preserved ejection fraction
ECG	electrocardiography
DM	diabetes mellitus
mTOR	mechanistic target of rapamycin
GC/MS	gas chromatography–mass spectrometry

## Appendix A

Metabolic connections between BCAAs (leucine, isoleucine, and valine) and selected amino acid–related metabolites (2-hydroxybutyrate [2-HB]) and related amino acids (alanine and glycine).

Valine is exclusively glucogenic and associated with fatty acid uptake and insulin resistance. Leucine is strictly ketogenic, yielding acetyl-CoA and acetoacetate for ketone body production. Isoleucine is both glucogenic and ketogenic, producing acetyl-CoA and succinyl-CoA. The amino groups from BCAA transamination are transferred to alanine, which mediates nitrogen transport from muscle to liver via the alanine (Cahill) cycle. In parallel, 2-HB is produced from a-ketobutyrate derived from methionine and threonine metabolism, particularly under conditions of increased glutathione synthesis. Although 2-HB arises from distinct pathways, these pathways share upstream metabolic stressors, including mitochondrial dysfunction, an increased NADH/NAD* ratio, oxidative stress, and insulin resistance, which can lead to the simultaneous elevation of the metabolite. 2-HB, 2-Hydroxybutyric acid; ALT: Alanine aminotransferase; BCAA, Branched-chain amino acids; BCAT, Branched-chain aminotransferase; BCKA, Branched-chain α-keto acid; BCKDH, Branched-chain α-keto acid dehydrogenase; CoA, Coenzyme A; HMG-CoA, 3-Hydroxy-3-methylglutaryl-CoA; TCA cycle, Tricarboxylic acid cycle.
