# Supplementary material for: Association Between Serum Leucine and NT-proBNP Levels in Relation to Fragmented QRS: A Multiomic Analysis of the HOZUGAWA Cohort
Source: Nutrients. 2026 Jul 11;18(14):2271. doi: 10.3390/nu18142271 (PMC13415236; doi:10.3390/nu18142271)
Supplement: Supplementary file 1 [file nutrients-18-02271-s001.zip › nutrients-4360672-supplementary.pdf]

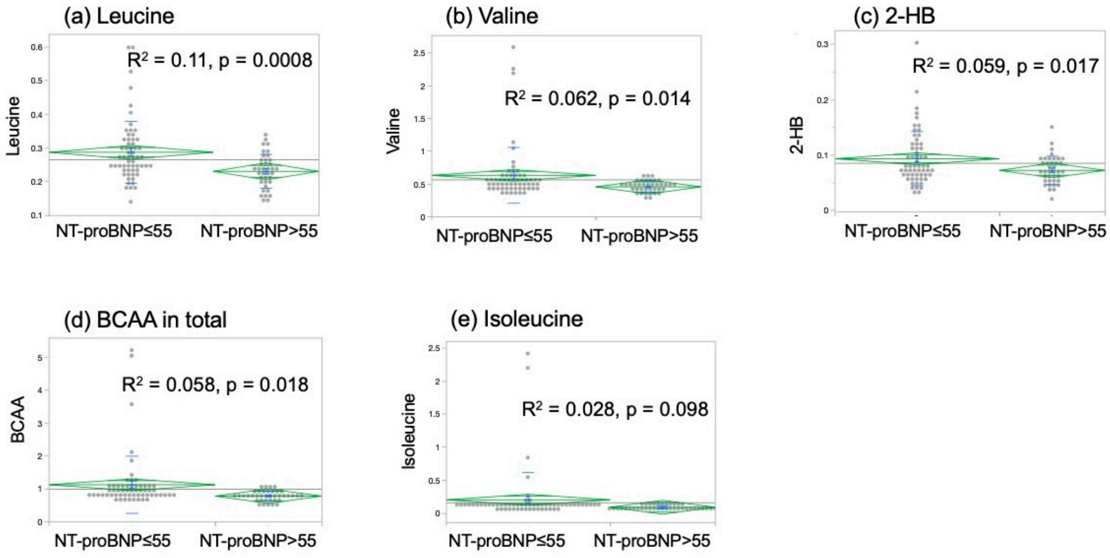


Supplementary Figure S1. Differences in serum BCAA and selected amino-acid–related metabolites according to NT-proBNP levels in participants with fQRS

Comparisons of relative serum levels of BCAAs (leucine, isoleucine, valine, and total BCAAs) and 2-HB between participants with NT-proBNP > 55 pg/mL and those with NT-proBNP ≤ 55 pg/mL. Statistical significance was assessed using appropriate group comparison tests.
